# Brainstem impairment in obstructive sleep apnoea and the effect of CPAP treatment: an electrophysiological blink reflex study

**DOI:** 10.1007/s11325-023-02944-8

**Published:** 2023-11-04

**Authors:** Claudio Liguori, Mariana Fernandes, Matteo Spanetta, Martina Zanovello, Maria Pia Giambrone, Clementina Lupo, Fabio Placidi, Francesca Izzi, Nicola Biagio Mercuri, Mariangela Pierantozzi

**Affiliations:** 1 Sleep Medicine Centre, University Hospital of Rome ‘Tor Vergata’, Rome, Italy; 2 Neurology Unit, University Hospital of Rome ‘Tor Vergata’, Rome, Italy; 3 Department of Systems Medicine, University of Rome ‘Tor Vergata’, Rome, Italy

**Keywords:** Blink reflex, Sleep impairment, Nocturnal hypoxia, Brainstem, Therapy

## Abstract

**Purpose:**

This study aimed to evaluate the functionality of the brainstem structures through the blink reflex (BR) test in patients with obstructive sleep apnoea (OSA) and to assess the effects of continuous positive airway pressure (CPAP) treatment on BR responses.

**Methods:**

Patients with moderate-severe OSA and controls underwent BR testing. Patients with OSA who were adherent to CPAP therapy repeated BR testing at 6 months follow-up. CPAP adherence was defined as CPAP use for ≥ 4 hour per night on > 5 nights per week with residual apnoea-hypopnea index less than 5 events per hour.

**Results:**

A total of 22 patients with OSA (86% male, mean age 57.8 ± 10.6 years) and 20 controls (60% male, mean age 55.3 ± 9.3 years) were included. Patients with OSA showed longer right and left R1 latency, as well as delayed right ipsilateral and contralateral R2 latencies compared to controls. Patients with OSA who were compliant with CPAP treatment (*n* = 16; 88% men, mean age 58.8 ± 9.7 years) showed a significant decrease in latency of the right ipsilateral and contralateral R2 responses at 6 months.

**Conclusion:**

This study showed an abnormal pattern of BR responses in patients with OSA, consistent with a significant impairment of brainstem functionality in OSA. CPAP treatment partially improved the BR responses, suggesting the importance of treating OSA.

## Introduction

Obstructive sleep apnoea (OSA) is a common sleep disorder affecting all ages and is associated with several systemic and neurologic consequences [1, 2]. OSA can impair brain function since it may exert a negative effect on cognitive performance, trigger neuroinflammation and neurodegenerative processes, and alter brain network functioning [3–8]. Over the years, neurophysiological studies have been carried out aimed at identifying possible central nervous system alterations related to OSA. In this context, evoked potentials and electroneurography-based studies revealed deficits in synaptic signalling and cognitive performance, as well as impairment of both motor and sensory peripheral nerve conduction in patients with OSA. However, no definitive results have been formulated and further studies should be performed to assess the neurophysiological consequences of OSA. Among the different electrophysiological exams, the blink reflex (BR) allows to explore the brainstem structure. BR is currently clinically used to detect both abnormalities of the peripheral trigeminal and facial nerves and brainstem central lesions.

Although several brainstem neural networks exert a crucial role in sleep and respiratory control and it has been supposed to be involved in the pathophysiology of sleep-related breathing disorders, to date, studies investigating the BR responses in OSA are few and somewhat inconclusive [9, 10]. Therefore, the present study aimed to explore the possible involvement of brainstem structures in patients with moderate-severe OSA (apnoea-hypopnea events ≥ 15 per hour of sleep) by using the BR responses to evaluate the effect of this sleep disorder on brainstem structures. Furthermore, considering that continuous positive airway pressure (CPAP) is the gold standard therapy for OSA, this study also aimed to investigate the effects of CPAP on BR responses in patients with OSA.

## Methods

### Participants

The study enrolled patients with moderate-severe OSA defined by an apnoea-hypopnea index (AHI) ≥ 15 events per hour of sleep at the Sleep Medicine Centre of the University Hospital of Rome Tor Vergata, as well as a control group of healthy subjects similar in age and sex to patients with OSA. All participants underwent physical and neurological examinations, a standard sleep medicine visit, including the administration of the Epworth Sleepiness Scale (ESS), and the BR recording. Only patients with OSA performed the overnight polygraphy.

Patients included in the study needed to meet the following inclusion criterion: diagnosis of moderate-severe OSA (AHI ≥ 15/h), confirmed by the polygraphic recording, and according to AASM criteria [11, 12]. For the control group, the inclusion criterion was the absence of sleep disorders, evaluated with a thorough structured sleep medicine interview and validated sleep questionnaires. The exclusion criteria for patients and controls were the following: heavy smoking, bronchial asthma, chronic obstructive pulmonary disorders, and interstitial lung diseases, autoimmune disorders, malignancies diabetes, stroke, hypertension and/or history of hypertensive crisis, and concomitant psychiatric or neurological disorders. Moreover, participants had to show current systolic blood pressure < 140 mmHg, diastolic blood pressure < 85 mmHg, and fasting blood glucose < 100 mg/dL.

All patients with OSA started CPAP treatment after the baseline evaluation and were followed at the Sleep Medicine Centre. The CPAP efficacy and compliance were both documented by the ventilator software report, and good compliance with CPAP treatment was established according to previously published criteria: CPAP use for ≥ 4 h per night and > 5 nights per week [13–15]. Only those patients with OSA who were documented to have good compliance and an AHI < 5 per hour, as confirmed by polygraphic recording at the 6-month follow-up visit, were included in the follow-up evaluation and repeated the BR recording.

Patients and controls provided their informed consent to the study, which was approved by the Independent Ethical Committee of the University Hospital of Rome “Tor Vergata.”

### Polygraphic recording and daytime sleepiness evaluation

Polygraphic recordings were performed using a validated instrument (Embletta; Embla, Amsterdam, The Netherlands). The following parameters were collected: AHI, defined as the sum of all apnoeas (> 90% reduction in airflow for > 10 s) and all hypopneas (> 30% reduction in airflow > 10 s) associated with ≥ 3% O_2_ desaturation [11]; mean oxygen saturation (SaO2), lowest SaO_2_, time spent with SaO_2_ < 90% (*T* < 90), and oxygen desaturation index (ODI) (number of oxygen desaturations ≥ 3% per hour). Daytime sleepiness was assessed through the ESS, administrated at baseline and after 6 months of CPAP treatment in patients with OSA [16, 17].

### BR recordings

The BR is a neurophysiological exam investigating the entire reflex arc linking the fifth (trigeminal) to the seventh (facial) cranial nerve by their synaptic connections in the pons and medulla. It consists of two components: the early R1, ipsilateral to the stimulated side, which expresses the disynaptic trigeminal-facial reflex connecting the pontine main nucleus of the fifth nerve and the ipsilateral nucleus of the seventh nerve; and the late bilateral R2 response, produced by a multisynaptic pathway involving the spinal tract of the fifth and the ipsilateral and contralateral facial nuclei [18, 19].

The BR was recorded in a quiet room, with participants comfortably lying supine with their mouths slightly open and eyes open. Each supraorbital nerve was stimulated with a bar electrode (the cathode over the supraorbital foramen and the anode 2 cm rostrally). Surface recording electrodes were placed on the inferior part of each orbicularis oculi muscle, the reference electrodes were lateral to the lateral cantus and the ground electrode was located on the chin. To obtain the R1 response, ipsilateral to the stimulated supraorbital nerve, and the bilateral R2 responses, recordings from both orbicularis oculi muscles were performed contemporarily by a two-channel recording system [18, 19].

The recording setting was based on stimuli of 0.1 ms duration with intensity 5–10 mA, applied via the supra-orbital margin. Signals were amplified and filtered (bandwidth 20–2000 Hz). Interstimulus intervals of 7 s were used to prevent habituation; 5–10 responses per site were obtained and superimposed to identify the shortest response latencies of ipsilateral R1 and bilateral R2 responses.

To ensure the reliability of results, the physicians involved in BR recordings (MPG, MZ, MS), as well as in data analysis (MP) were completely blind to the participant’s condition.

### Statistical analysis

Descriptive statistics were computed to characterize the sample in terms of demographic and clinical data. The normality of the data was assessed through the Shapiro–Wilk test. Mann–Whitney *U* tests were used to compare the demographic, clinical, polygraphic cardiorespiratory data, and BR responses between patients with OSA and controls. For the longitudinal analyses, differences in BR latencies at baseline and at the 6-month follow-up in patients with OSA compliant with CPAP were tested using the Wilcoxon signed rank test. The *p*-value was set at *p* < 0.05 for statistical significance. The statistical analysis was performed using commercial software SPSS version 25 [20].

## Results

A total of 22 patients affected by OSA (86.4% men; mean age of 57.8 ± 10.6 years), and 20 healthy controls similar in sex and age (60.0% men; mean age of 55.3 ± 9.3 years) were included in the study. Demographic, clinical, and polygraphic features of both groups are summarized in Table 1. Furthermore, the differences in BR response latencies between patients with OSA and controls are displayed in Table 2.

**Table 1:** Clinical and polygraphic data of patients and controls

	Patients with OSA (*n* = 22)	Controls (*n* = 20)	*p*-value
Sex			
Men	19 (86.4%)	12 (60.0%)	0.11
Women	3 (13.6%)	8 (40.0%)	
Mean age in years	57.8 ± 10.6	55.3 ± 9.3	0.33
BMI	28.0 ± 3.4	25.9 ± 2.1	0.05
AHI	38.3 ± 17.3	NA	
ODI	36.3 ± 22.1	NA	
Mean SaO_2_ (%)	93.3 ± 1.5	NA	
ESS	11.6 ± 4.2	NA	

*Abbreviations*: *BMI* body mass index, *AHI* apnoea-hypopnea index, *ODI* oxygen saturation index, *NA* not applicable, *Mean SaO*_*2*_ mean oxygen saturation, *ESS* Epworth Sleepiness Scale

**Table 2:** Blink Reflex test data in OSAS patients and controls

	Patients with OSA (*n* = 22)		Controls (*n* = 20)		Mann–Whitney test	
	Median	25–75th percentiles	Median	25–75th percentiles	U	*p*-value
Blink test						
R1 left	12.0	11.3–12.2	11.2	10.1–11.9	132.5	0.03
R2 left ipsilateral	34.3	31.1–36.8	31.9	29.3–34.2	162.5	0.15
R2 left contralateral	35.9	29.9–39.1	32.00	31.6–34.6	148.0	0.07
R1 right	12.1	11.6–12.7	10.6	9.8–11.8	82.0	0.001
R2 right ipsilateral	33.5	31.9–35.8	32.1	30.6–33.6	142.0	0.049
R2 right contralateral	34.5	31.8–38.1	33.2	31.0–33.8	119.5	0.01

Sixteen patients with OSA who were compliant with CPAP therapy at the 6-month follow-up were included in the longitudinal analysis (87.5% men, mean age of 58.8 ± 9.7 years). The latency of the right ipsilateral R2 response and the right contralateral R2 response significantly decreased from baseline to follow-up (see Table 3).

**Table 3:** Blink reflex test at baseline and at 6-month follow-up in the subgroup of patient with OSA compliant with CPAP treatment (*n* = 16)

	Patients with OSA compliant with CPAP treatment (*n* = 16)					
	Baseline		6-month follow-up		Wilcoxon test	
	Median	25–75th percentiles	Median	25–75th percentiles	*Z*	*p*-value
AHI	32.8	26.5–47.1	2.4	1.1–4.6	− 3.516	< 0.001
ESS	12.0	9.0–15.0	7.0	7.0–10.5	22.00	0.01
Blink test						
R1 left	12.0	11.3–12.5	11.8	11.2–12.4	− 1.010	0.31
R2 left ipsilateral	34.3	31.4–37.0	33.0	30.2–35.6	− 1.862	0.06
R2 left contralateral	36.5	31.9–39.3	35.3	29.7–38.4	− 1.552	0.12
R1 right	12.05	11.5–12.6	11.7	11.1–12.5	− 1.063	0.29
R2 right ipsilateral	33.5	32.3–35.9	31.9	30.0–33.7	− 2.588	0.01
R2 right contralateral	35.8	33.3–38.3	33.3	28.6–33.7	− 3.362	0.001

*Abbreviations*: *AHI* apnoea-hypopnea index, *ESS* Epworth Sleepiness Scale

## Discussion

The main result of this study was the impairment of BR responses in patients with OSA compared to controls. Our results showed in these patients a prolonged latency of R1 response after stimulation of both supraorbital nerves and a delayed ipsilateral and contralateral latency of R2 response after stimulation of the right supraorbital nerve. The alteration of BR reported in this study may be considered as the electrophysiological evidence of brainstem dysfunction associated with the OSA condition. Although our findings demonstrated a BR response impairment in patients with moderate-severe OSA, the mechanisms at the basis of these results can only be hypothesised. Delayed BR responses may, in fact, reflect an altered brainstem neurotransmission due to the impaired myelination of cranial nerves and dysregulation of brainstem synaptic pathways, both related to hypoxia and sleep impairment [21]. Considering that R1 is thought to represent the disynaptic reflex connecting the main trigeminal sensory nucleus to the ipsilateral facial nucleus, the delayed R1 response recorded in patients with OSA seems to suggest the involvement of synaptic relay stations sited in the mid pons and lower pontine tegmentum. The lesser implication of R2 than R1 responses may indicate a greater preservation of the multisynaptic pathway linking the spinal tract of the trigeminal nucleus to the facial nucleus, as specifically investigated by the R2 response, which could be interpreted as a minor involvement of medulla oblongata structures in OSA. Therefore, the BR pattern abnormalities found in patients with moderate-severe OSA allow us to hypothesise that this sleep disorder may be associated with impairment in structures mainly present in the pons, which is a crucial region for the sleep–wake cycle regulation and is also a region that holds the main nuclei involved in the sleep and respiratory control, namely, the Medial parabrachial/Kolliker Fuse, Lateral parabrachial and GABAergic and glutamatergic ventral pons regions [22–24]. Conversely, the mainly respiratory nuclei located in the medulla oblongata, comprising the ventral respiratory neurons associated with the nucleus ambiguous and the dorsal respiratory neurons (DRN) associated with the nucleus tractus solitarius [25], seem to be less affected in OSA due to the partial preservation of the conduction of R2 responses. On these bases, it could be speculated that pontine structures are more vulnerable to a possible OSA-related intermittent nocturnal hypoxemia effect than the medulla centres, with a further dysfunction of pontine reticular pathways in patients with OSA.

Few studies have investigated the effects of OSA on BR responses with mixed results [26–29]. In line with the present findings, the study by Urban et al. (1996), although not showing significant changes in electrophysiological brainstem responses in a group of 18 patients with OSA, documented abnormalities in the left BR R1 component in one patient, suggesting a left pontine lesion [10]. However, more recently, Tavsanli and colleagues found no significant differences in BR response latencies between patients with OSA and controls [30]. Considering the paucity of studies investigating the BR responses in patients with OSA, further studies are needed for reaching a unequivocal conclusion, that currently cannot be postulated. However, the present data, coupled with previous studies based on evoked potentials and peripheral nerve conduction recordings, may suggest the impairment in electrophysiological conduction in patients with OSA, and that it can be possibly attributed to a hypoxic mechanism inducing myelin damage and axonal alteration [31–37]. In particular, visual and auditory evoked potentials studies showed prolonged latency and reduced amplitude of the evoked responses [36, 37], hypothesizing a myelin damage due to intermittent hypoxia and neuro-inflammation based on delayed latency, and the occurrence of microvascular events to explain the reduced amplitude.

Considering that CPAP is the gold standard treatment for patients with moderate-severe OSA [38, 39], the present study evaluated the effects of beneficial CPAP therapy on the BR documenting a partial improvement of responses with the reduction of the delayed ipsilateral and contralateral R2 latency after the right supraorbital nerve stimulation. No other significant modifications of the BR responses were observed, in particular in the R1 latencies. These data, showing the persistent alteration of the R1 responses despite the effective CPAP therapy, seem to confirm the greater vulnerability of the pontine structures compared to those of the medulla towards a possible hypoxic effect, which remains stable although the significant improvement of the AHI following CPAP treatment. Nonetheless, it is important to note that the oxygen saturation parameters were evaluated exclusively at the polygraphic recording performed at baseline, while at the 6-month follow-up, the AHI was monitored through the software report. Therefore, although the CPAP effect on the AHI, it is not possible to disentangle whether the lack of improvement in the R1 response can be related to the susceptibility of the pontine structure to the chronic suboptimal oxygen levels. To our knowledge, no previous study had explored the effects of CPAP treatment on BR responses in OSA, and thus, the comparison of the present results with previous investigations cannot be performed. However, a study performed on severe OSA and evaluating the effect of CPAP therapy on the auditory evoked potentials did not document an improvement of electrophysiological responses after treatment, suggesting an un-modifiable effect of OSA on the pontine regions [26] and not replicating previous results [29]. Taking into account the whole literature about the effects of OSA on the electrophysiological tests and the possible beneficial effect of CPAP treatment, there are studies documenting the significant improvement of optic nerve function and neuropsychological event-related potentials in patients with OSA treated by CPAP, thus highlighting the importance of performing further studies with a larger group of patients to test this effect [26–29].

This study presents some limitations that need to be addressed. The diagnosis of OSA was performed by using polygraphic cardiorespiratory monitoring, which did not allow the evaluation of sleep structure. Furthermore, the inclusion and exclusion criteria were restricted, which allowed the inclusion of a homogeneous sample of patients not affected by comorbidities, however on the other hand limited the sample size, possibly affecting the BR statistical power.

In conclusion, considering the detrimental effects of OSA on brain functions, as reported by previous electrophysiological studies, this BR study demonstrated that OSA can impair brainstem functioning, mostly involving the mid-pons region. Hence, the present study underlines the importance of early recognition of OSA and thus starting CPAP therapy in order to preserve and recover brain and brainstem network functions. The application of electrophysiologic tools exploring brainstem functions, such as BR, may be proposed for the assessment of OSA as an instrument that may improve the comprehension of pathophysiologic mechanisms and clinical outcomes of this common sleep disorder.

## Data Availability

The dataset generated during the current study is available from the corresponding author on reasonable request.

## Abbreviations

OSA: Obstructive sleep apnoea
BR: Blink reflex
CPAP: Continuous positive airway pressure
AHI: Apnoea-hypopnea index
ESS: Epworth Sleepiness Scale
